# Hemosuccus pancreaticus post-EUS-FNA in multicystic pancreatic tail formation: A case report

**DOI:** 10.1097/MD.0000000000041453

**Published:** 2025-02-07

**Authors:** Goran Bokan, Eva Mislej, Borut Štabuc, Darko Siuka

**Affiliations:** a Department of Gastroenterology and Hepatology, Internal Medicine Clinic, University Clinical Centre of the Republic of Srpska, Banja Luka, Bosnia and Herzegovina; b Faculty of Medicine, University of Banja Luka, Banja Luka, Bosnia and Herzegovina; c Department of Gastroenterology, University Medical Center Ljubljana, Ljubljana, Slovenia; d Medical Faculty Ljubljana, University of Ljubljana, Ljubljana, Slovenia.

**Keywords:** EUS-FNA, gastrointestinal bleeding, hemosuccus pancreaticus, pancreatic cyst, wirsungorrhagia

## Abstract

**Rationale:**

Hemosuccus pancreaticus (HP) is a rare but serious cause of gastrointestinal bleeding, characterized by hemorrhage from the papilla of Vater via the pancreatic duct. It is typically associated with vascular or pancreatic pathologies, including pancreatic cysts, tumors, or pseudoaneurysms involving adjacent arteries such as the splenic or gastric artery.

**Patient concerns:**

A 68-year-old male patient was evaluated for a pancreatic tail cyst detected on magnetic resonance cholangiopancreatography (MRCP). The lesion was described as a 20-mm multilocular cystic formation with septations, raising suspicion of malignancy.

**Diagnosis:**

HP was diagnosed following an episode of gastrointestinal bleeding after endoscopic ultrasound-guided fine-needle aspiration (EUS-FNA) of the pancreatic tail cystic lesion.

**Interventions:**

The patient underwent EUS-FNA via a transgastric approach to obtain a tissue sample for further evaluation.

**Outcomes:**

The procedure was complicated by gastrointestinal bleeding and later confirmed to be HP. The bleeding was managed conservatively, and the patient remained hemodynamically stable with spontaneous resolution of symptoms.

**Lessons:**

This case underscores a rare but significant complication of EUS-FNA. Given the limited number of reported cases, further research is needed to establish the incidence, risk factors, and preventive measures for HP in similar clinical scenarios.

## 1. Introduction

Hemosuccus pancreaticus (HP) is defined as bleeding from the papilla of Vater through the pancreatic duct. The term itself was first proposed by Sandblom in 1970. Historically, there have been several terms used to describe the same condition, including wirsungorrhagia by Van Kemmel in 1969 and hemowirsungia.^[1]^

The bleeding is caused by a source in the pancreas, pancreatic duct, or structures adjacent to the pancreas, such as splenic or gastric artery. It is most commonly caused by acute or chronic pancreatitis. However, in rare cases, it can be a symptom of pancreatic tumors or pancreatic cystic neoplasms.^[1,2]^

The age of onset is widely distributed. According to the literature, men are more prone to HP than women.^[3]^

HP usually presents as upper gastrointestinal bleeding with melena or, less frequently, hematemesis. It can also present as intra-abdominal or retroperitoneal bleeding. Due to blood clot formation in the pancreatic duct, which causes obstruction, patients could also experience colicky abdominal pain. The bleeding is usually intermittent and not severe enough to cause hemodynamic instability, usually only causing slight abnormalities in blood tests.^[1,2]^

The diagnosis is challenging due to its rarity. It could be diagnosed through direct endoscopy although active bleeding from the papilla of Vater is seen in only about 30% of the cases.^[4]^

The vast majority of the bleeding is managed nonsurgically, usually by interventional radiographic procedures, which have an overall success rate of 67%. For patients with hemodynamic instability, a surgical approach is inevitable.^[2,5]^

## 2. Case report

A 68-year-old male patient presented for an elective endoscopic ultrasound-guided fine-needle aspiration (EUS-FNA) of a pancreatic tail cyst, which was verified by magnetic resonance cholangiopancreatography. The cyst measured 20 mm in diameter and was described as a multilocular cystic formation with septations, which did not cause pancreatic duct dilatation. Although a simple cyst was suspected, the possibility of malignant intrapancreatic neoplasia could not be ruled out. The patient had no subjective complaints at the time of the procedure. His medical history included prostate cancer, basal cell skin cancer, and superior mesenteric artery dissection. He was on chronic antihypertensive therapy.

**Figure 1.**
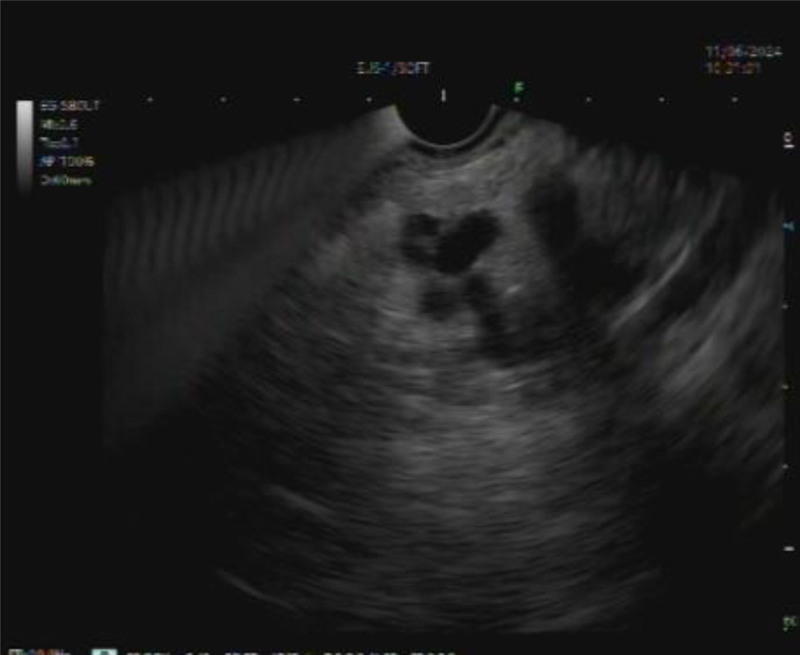
A multilocular cystic formation in the tail of the pancreas visualized by EUS.

Due to suspicion of potential malignancy and previous medical history, we indicated a biopsy. The EUS-FNA was performed via the transgastric route. At the transition of the body and tail of the pancreas, an 18-mm cystic formation was observed and punctured with a Boston Scientific Expect 22G needle (Fig. 1). We did not notice any communication between the cyst and the main pancreatic duct on EUS. The string sign during the procedure was positive. The sample was sent for laboratory determination of carcinoembryonic antigen, which could not be determined, because the aspirated sample was very dense and tough.

**Figure 2.**
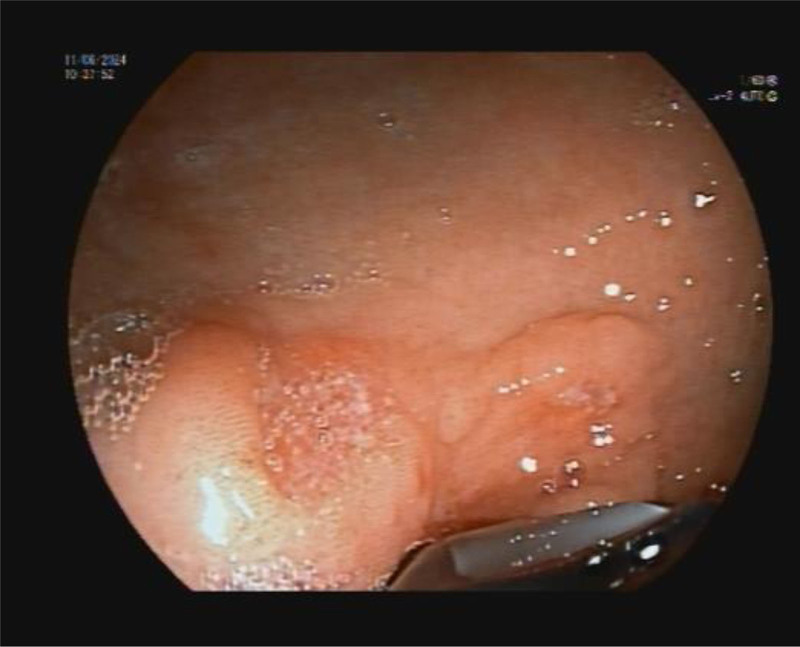
A minor ulceration localized in the area of the antrum of the stomach.

**Figure 3.**
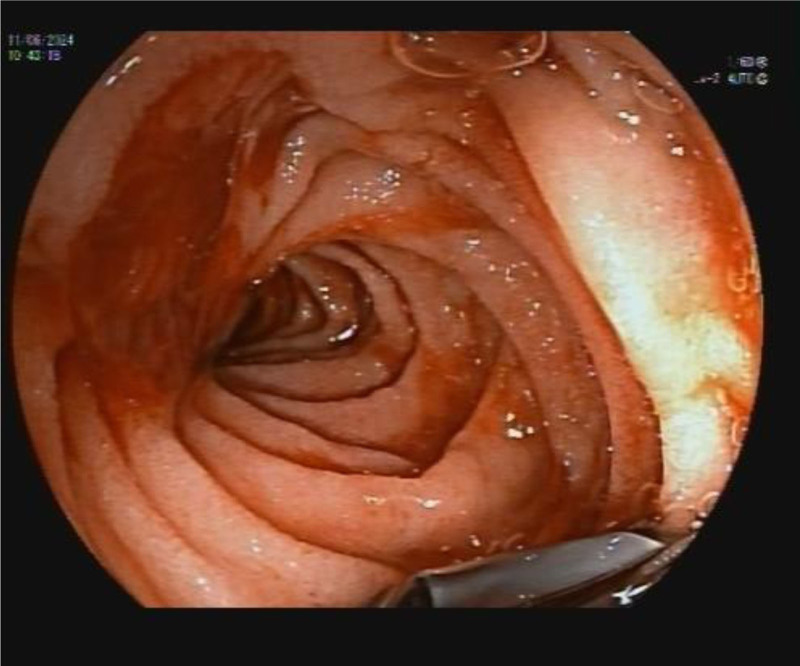
Bleeding on the major duodenal papilla after placement of the endoscopic device in the duodenum, so-called wirsungorrhagia.

Also, the cyst fluid cytology of the sample was performed, which showed the contents of a cystic formation in which there were neither epithelial nor malignant cells. During the examination, an 8-mm ulcer was observed in the antrum of the stomach (Fig. 2). After passing with the endoscope to the duodenum, bleeding from the major duodenal papilla was observed (Fig. 3). The decision was made to hospitalize the patient for monitoring of his blood count and general condition.

**Figure 4.**
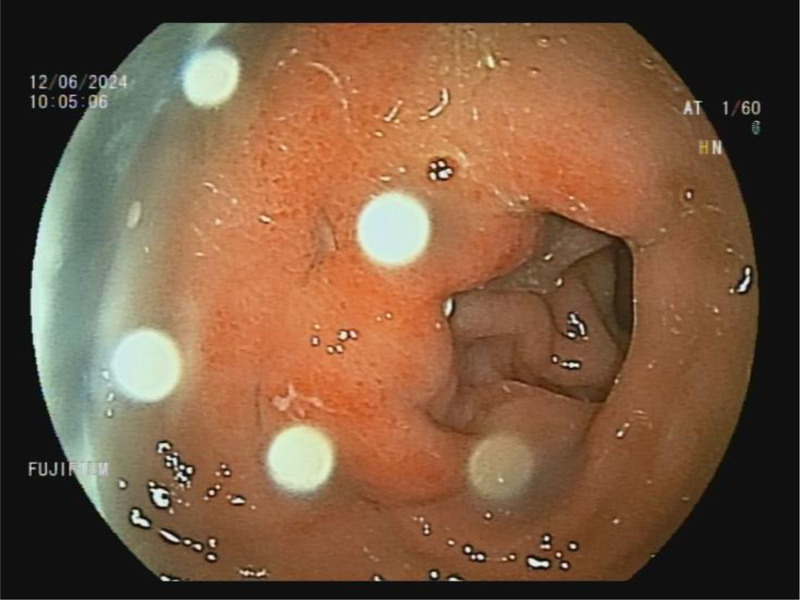
A control gastroscopy performed the next day, where 2 ulcerations were noted in the antrum of the stomach.

The next day, an esophagogastroduodenoscopy was repeated, revealing the previously described gastric antrum ulcer, as well as 2 smaller ulcers in the duodenal bulb (Fig. 4). Multiple biopsies of the ulcerations and gastric mucosa were performed, which described the signs of chronic gastritis/healing ulcer caused by helicobacter pylori infection. After the administration of a parenteral antispasmodic drug hyoscine butyl bromide during the esophagogastroduodenoscopy, a small amount of fresh blood flowing from the papilla of Vater was observed. The patient remained hemodynamically stable throughout, without significant deviations in the hemogram or other laboratory-biochemical findings, also without abdominal pain or discomfort. He was subsequently discharged after 1 night of hospitalization for further home treatment and helicobacter pylori eradication therapy.

Considering the absence of malignant features and that this is most likely a simple multilocular cystic formation, we have decided to monitor it with EUS in 6 to 12 months and adjust the plan accordingly based on its dynamics.

## 3. Discussion

EUS-FNA is generally considered a safe procedure, with a well-defined low complication rate. Intracystic bleeding post-procedure may occur; however, it rarely results in HP.

The importance of the cyst and main pancreatic duct relationship should be discussed. There are 2 potential pathogenesis of HP. The first is the intracystic bleeding in a cyst with communication with the main pancreatic duct (eg, in intraductal papillary mucinous neoplasm) with the passage of blood from the cyst into the main pancreatic duct. In these cases, the EUS procedure and the device employed can impact HP onset. For example, using microforceps to perform a through-the-needle biopsy of the pancreatic cyst wall increases the risk of intracystic bleeding.^[6]^ The second pathogenesis is a direct main pancreatic duct injury with the needle during EUS-FNA of a hypervascular cyst (eg, cystic neuroendocrine tumor or serous cystadenoma) with the passage of blood from the tumor into the main pancreatic duct through the needle trajectory.^[7]^

There are only a few cases documented in the literature, likely because the bleeding itself usually does not produce significant symptoms and upper endoscopy is generally not performed. A PubMed search using the terms “EUS-FNA,” “pancreatic cyst,” and “hemosuccus pancreaticus” yielded 3 relevant case reports in which HP occurred after EUS-FNA. There are, however, more case reports of HP in patients with acute or chronic pancreatitis and in those with splenic aneurysms.

Mohamadnejad et al^[8]^ described 2 cases of HP. In both instances, the bleeding was successfully stopped with the EUS application of cyanoacrylate glue to the splenic artery pseudoaneurysm. There are not many case reports described, where cyanoacrylate glue is used to obliterate splenic pseudoaneurysm.

The 3 published case reports that have described HP following EUS-FNA were written by Singh et al,^[9]^ Cheruvattath et al^[10]^ (which is actually a response to Singh et al), and Keswani.^[11]^ The case report by Keswani^[11]^ describes a blood aspirate after the first passage with a 19G needle. A repeat EUS-FNA of a separate component of a cyst yielded clear aspirate. The EUS was performed again, which revealed hyperechoic filling of a pancreatic duct consistent with blood. Their patient developed mild pancreatitis afterward but was discharged without any serious complications.^[11]^

What differentiates this case from the others is the bloody aspirate that was seen after the first passage with a needle, which, in our opinion, strongly suggests that HP in this instance was a result of the first needle passage.

The histology in their case was consistent with that of a serous cystadenoma, which is considered to have a low-risk rate for HP according to the literature. In the case report written by Singh et al,^[9]^ the aspirate was clear. They only did 1 passage with a 19G needle and observed HP immediately after the puncture. Repeat imaging of the cyst by EUS revealed hyperechoic material in the cyst, confirming intracystic bleeding. In our case, an imaging of the cyst, which was done immediately after observing HP, did not show any significant findings.

In the case report written by Cheruvattath et al,^[10]^ a 22G needle was used, as well as in our case, and it was suggested that the bleeding might have been caused by rapid needle decompression. However, a 19G needle was used in the other 2 cases, which also resulted in HP. This suggests that the needle size may not be a significant factor although observational studies are needed to draw definitive conclusions. According to our literature review, the bleeding is also independent of the site of the puncture. The cyst of our patient was located in the tail of the pancreas, which is notable because the blood had to traverse the entire length of the pancreas as also described by Cherruvattath et al.^[10]^ In our case, HP was observed during the initial procedure, which is typically the scenario described in the literature.^[9]^ Our patient did not undergo a CT scan, which is a limitation of our case. We did not perform the CT scan because the bleeding stopped spontaneously, and the general condition of the patient was good. In all of the cases discussed, including ours, the patients were discharged without any complications of HP.

## 4. Conclusion

Even though HP after EUS-FNA is a rare entity, it should be considered, especially if the patient develops symptoms of upper gastrointestinal bleeding after the procedure. The majority of the cases can, however, be managed conservatively.

The literature contains only a limited number of case reports, and there are no observational studies available. That is why the actual risk rate of HP following EUS-FNA is unknown.

Further research is warranted to better understand the incidence and risk factors for HP after EUS-FNA.

## Author contributions

**Conceptualization:** Goran Bokan, Eva Mislej, Darko Siuka

**Formal analysis:** Goran Bokan, Darko Siuka

**Funding acquisition:** Goran Bokan

**Investigation:** Goran Bokan

**Methodology:** Goran Bokan, Eva Mislej

**Visualization:** Goran Bokan

**Writing – original draft:** Goran Bokan, Eva Mislej

**Supervision:** Borut Štabuc, Darko Siuka

**Writing – review & editing:** Borut Štabuc

**Validation:** Darko Siuka
